# *Spiroplasma ixodetis* in Ticks Removed from Humans, Sweden and Åland Islands, Finland

**DOI:** 10.3201/eid3111.250545

**Published:** 2025-11

**Authors:** Malin Lager, Yousif Alkattan, Amanda Sandbacka Karlsson, Louise Fernström, Anna Grankvist, Christine Wennerås, Marika Nordberg, Dag Nyman, Per-Eric Lindgren, Pia Forsberg, Peter Wilhelmsson, Anna J. Henningsson

**Affiliations:** National Reference Laboratory for Borrelia and Other Tick-Borne Bacteria, Jönköping, Sweden (M. Lager, P.-E. Lindgren, P. Wilhelmsson, A.J. Henningsson); Linköping University, Linköping, Sweden (Y. Alkattan, A. Sandbacka Karlsson, P.-E. Lindgren, P. Forsberg, P. Wilhelmsson, A.J. Henningsson); Lycksele Hospital, Region Västerbotten, Sweden (L. Fernström); National Reference Laboratory for Borrelia and other Tick-Borne Bacteria, Gothenburg, Sweden (A. Grankvist, C. Wennerås); Sahlgrenska Academy at the University of Gothenburg, Gothenburg (C. Wennerås); Åland Health Services, Mariehamn, Finland (M. Nordberg); The Borrelia Research Group of the Åland Islands, Mariehamn (M. Nordberg, D. Nyman); European Society for Clinical Microbiology and Infectious Diseases Study Group for Tick-Borne Diseases, Basel, Switzerland (D. Nyman, P.-E. Lindgren, A.J. Henningsson)

**Keywords:** Spiroplasma ixodetis, prevalence, spiroplasmosis, coexistence, vector-borne infections, bacteria, ticks, Sweden, Åland Islands, Finland

## Abstract

The prevalence of *Spiroplasma ixodetis* in ticks that have bitten humans in Sweden and in the Åland Islands, Finland, was 2.6%, with observed significant geographic differences between regions. The pathogen was not detected in blood samples from participants bitten by *S. ixodetis*–positive ticks, indicating low risk for transmission to humans.

*Spiroplasma ixodetis*, an emerging tickborne bacterium transmitted by *Ixodes ricinus* ticks, shows a prevalence range of 0.4%–3.0% in questing ticks (*1*,*2*) to 13.5% in ticks removed from humans in Europe (*3*). Human cases of *S. ixodetis* infection, spiroplasmosis, have been reported from Europe, including Sweden (*4*,*5*), in both immunocompetent and immunosuppressed patients; symptoms were more severe in immunosuppressed patients (*4*,*6*).

Low awareness, nonspecific symptoms similar to other tickborne infections, and potential co-infection with other tickborne pathogens contribute to misdiagnosis or underdiagnosis (*7*,*8*). Because *S. ixodetis* is an intracellular bacterium, culturing is difficult, and no serologic assays are available; the primary detection tool is PCR. We assessed the risk for human exposure to this pathogen by investigating the prevalence, geographic distribution, and potential coexistence with other tickborne pathogens in feeding ticks removed from humans in Sweden and in the Åland Islands, Finland.

## The Study

This study is a part of the Tick-Borne Diseases (TBD) STING study, a prospective multicenter study in Sweden and in the Åland Islands, Finland. The study enrolled 2,327 healthy tick-bitten participants (>18 years of age) at primary healthcare centers (PHCs) in 4 geographic regions (*9*) (Figure) through public advertisements during 2008–2010. The Regional Ethics Review Board in Linköping, Sweden, and the Åland Health Care Ethics Committee approved the study.

**Figure:**
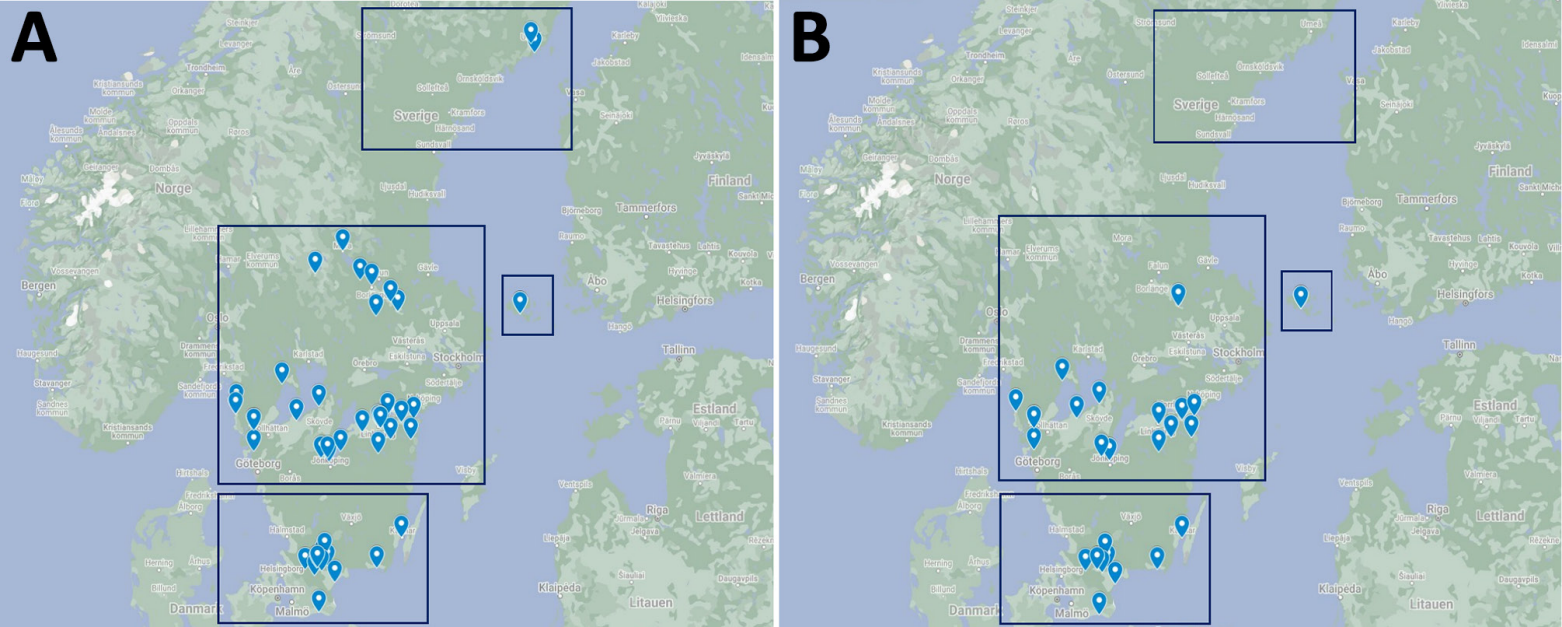
Locations of primary health care centers in study of *Spiroplasma ixodetis* in ticks removed from humans, Sweden and in the Åland Islands, Finland. Maps indicate all primary health care centers (A) and centers where *Spiroplasma ixodetis*–positive ticks were found (B) in 4 regions: northern Sweden (top boxes), southcentral Sweden (center left boxes), southernmost Sweden (bottom boxes), and the Åland Islands, Finland (center right boxes). Source: Google Maps (https://www.google.com/maps).

We homogenized tick specimens, extracted total nucleic acids, and reverse-transcribed them to cDNA (*9*) (Appendix). We also extracted DNA from blood plasma collected at inclusion (at the time of the tick bite) and at follow-up 3 months later from participants bitten by a *S. ixodetis*–positive tick. We detected *S. ixodetis* in ticks and plasma using a species-specific TaqMan real-time PCR targeting a 170-bp fragment of the RNA polymerase β subunit (*10*), then performed nucleotide sequencing and verification with BLAST (https://blast.ncbi.nlm.nih.gov/Blast.cgi). We analyzed all *S. ixodetis*–positive ticks for the presence of nucleic acid from *Borrelia burgdorferi* sensu lato, *B. miyamotoi*, tick-borne encephalitis virus, *Anaplasma phagocytophilum*, *Neoehrlichia mikurensis*, *Babesia* spp., and *Rickettsia* spp. by real-time PCR (*11*) (Appendix Table 1).

The study consisted of 2,735 *I. ricinus* ticks: 1,823 nymphs, 689 adults, 118 larvae, and 105 ticks for which we could not determine developmental stage (Table 1). The blood feeding time range was <24 to >72 hours (Appendix Table 2). Most (n = 1,156) ticks were collected in southcentral Sweden, followed by the Åland Islands (n = 950), southernmost Sweden (n = 605), and northern Sweden (n = 24) (Table 2). In total, 72 ticks (2.6%) were positive for *S. ixodetis*; of those, 60 showed >99.42% sequence identity with *S. ixodetis* strain sHm (GenBank accession no. AP026933.1). All plasma samples from study participants bitten by a *S. ixodetis–*containing tick yielded negative PCR results.

**Table 1:** Ticks testing positive for *Spiroplasma ixodetis*, by developmental stage, in study of *S. ixodetis* in ticks removed from humans, Sweden and Åland Islands, Finland

Developmental stage	Total no. (%)	No. (%) positive *
Larva	118 (4.0)	3 (2.5)
Nymph	1,823 (67)	44 (2.4)
Adult	689 (25)	22 (3.2)
F	654 (24)	21 (3.2)
M	35 (1.3)	1 (2.9)
Not determined†	105 (4.0)	3 (2.9)
Total	2,735 (100.0)	72 (2.6)

*Percentage calculated based on the number of *S. ixodetis*–positive ticks in relation to the total number of ticks in the study per developmental stage. †Developmental stage could not be determined because of damage to ticks during removal from host.

**Table 2:** Distribution of collected and *Spiroplasma ixodetis*–positive ticks, by geographic region, Sweden and in the Åland Islands, Finland

Region	Total no. (%)	No. (%) positive *
Southcentral Sweden	1,156 (42)	31 (2.7)
Åland Islands, Finland	950 (35)	4 (0.42)
Southernmost Sweden	605 (22)	37 (6.1)
Northern Sweden	24 (1.0)	0

*Percentage calculated based on the number of *S. ixodetis*–positive ticks in relation to the total number of ticks in the study per region.

We found no statistical differences in the prevalence of *S. ixodetis* among the different developmental stages of the ticks (Table 1); however, we found a statistically significant difference in geographic distribution of *S. ixodetis*–positive ticks between southcentral Sweden and southernmost Sweden (p = 0.0004), southcentral Sweden and the Åland Islands (p = 0.00005), and southernmost Sweden and the Åland Islands (p<0.00001). No ticks collected in northern Sweden were positive for *S. ixodetis.*

In total, 26 (36%) of the *S. ixodetis*–positive ticks contained >1 additional pathogens, mainly species in the *B. burgdorferi* s.l. complex (n = 14), of which most were *B. afzelii*. Coexistence of *S. ixodetis* and other tickborne pathogens was less common. Fifteen of the ticks with coexisting pathogens were nymphs, and 11 were adult females. Three ticks carried 3 pathogens: *S. ixodetis*, *B. burgdorferi* s.l., and *N. mikurensis* (Appendix Table 2). We found no statistically significant difference between observed frequency (17%) and expected frequency (19%) for coexistence between *S. ixodetis* and *B. burgdorferi* s.l. bacteria, suggesting that coexistence does not appear more often than expected by chance based on the prevalences of the individual pathogens (p = 0.738). We found statistically significant differences in geographic distribution of ticks with coexistence, regardless of pathogens, between southcentral Sweden and southernmost Sweden (p = 0.0004), southcentral Sweden and the Åland Islands (p = 0.04), and southernmost Sweden and the Åland Islands (p<0.00001).

## Conclusions

The overall prevalence of *S. ixodetis* in *I. ricinus* ticks removed from humans was 2.6%, with statistically significant differences in distribution between geographic areas. *S. ixodetis*–positive ticks were found in all developmental stages, including larvae, suggesting transovarial transmission of the bacterium. The prevalence in our study is consistent to previous studies showing a prevalence of 0.4%–3% in questing ticks (*1*,*2*). The number of ticks analyzed from northern Sweden was low, which can explain the negative results in this area. However, because of climate changes and raised temperatures, more suitable habitats for ticks and hosts might result in the spread of *S. ixodetis*–infected ticks into new areas (*12*). Few *S. ixodetis*–positive ticks were detected in the Åland Islands, an area with high tick density, highly endemic for *B. burgdorferi* s.l. and tick-borne encephalitis virus (*9*,*13*). That finding indicates that endemic areas for one pathogen may not be endemic for others.

The negative PCR results in plasma from participants who did not necessarily show symptoms related to tickborne diseases were consistent with a previous study (*3*). In that study, patients with influenza-like symptoms and erythema migrans also showed negative results in blood after being bitten by a tick carrying *S. ixodetis*. Even though *S. ixodetis* bacteria have been detected in blood (*4*), knowledge of the optimal time of sampling or the frequency of the pathogen in blood is limited. The inclusion sample was collected only days after the tick bite, perhaps before a potential bacteremia, and the follow-up sample was collected 3 months after a potential acute infection, which may be the reason for the negative outcome. No time lag for transmission has been established as of November 2025; because *S. ixodetis* is located in the midgut of the tick, we hypothesize that the time lag could be similar to that of *Borrelia* spp. transmission, 24–48 hours (*9*).

Most of the ticks carrying >1 pathogen contained both *S. ixodetis* and *B. burgdorferi* s.l. bacteria. That finding was not surprising because *Borrelia* spp. bacteria, mainly *B. afzelii*, are the most common pathogens found in questing ticks in Europe (*14*).

Our study used samples collected >15 years ago; although our findings might not reflect the current situation, they provide relevant epidemiologic insights into the prevalence and geographic distribution of *S. ixodetis*. Although we were unable to sequence all *S. ixodetis*–positive ticks, mainly because of high cycle threshold values (>35), we believe the real-time PCR findings are trustworthy because primers and probe are designed for species-specific detection.

Further studies are needed to expand our understanding of prevalence, geographic distribution, and the possibility of co-infection of tickborne *S. ixodetis*. Our results indicate low risk of being infected by *S. ixodetis* after a tick bite; however, spiroplasmosis and co-infections should be considered as differential diagnoses in cases of fever after a tick bite (*4*,*8*).

Appendix Additional information about *Spiroplasma ixodetis* in ticks removed from humans, Sweden and Åland Islands, Finland.

## References

[1] Olsthoorn F, Sprong H, Fonville M, Rocchi M, Medlock J, Gilbert L, et al. Occurrence of tick-borne pathogens in questing *Ixodes ricinus* ticks from Wester Ross, Northwest Scotland. Parasit Vectors. 2021;14:430. 34446082 10.1186/s13071-021-04946-5 PMC8393815

[2] Subramanian G, Sekeyova Z, Raoult D, Mediannikov O. Multiple tick-associated bacteria in *Ixodes ricinus* from Slovakia. Ticks Tick Borne Dis. 2012;3:406–10. 23182274 10.1016/j.ttbdis.2012.10.001

[3] Geebelen L, Lernout T, Tersago K, Terryn S, Hovius JW, Docters van Leeuwen A, et al. No molecular detection of tick-borne pathogens in the blood of patients with erythema migrans in Belgium. Parasit Vectors. 2022;15:27. 35057826 10.1186/s13071-021-05139-w PMC8772185

[4] Eimer J, Fernström L, Rohlén L, Grankvist A, Loo K, Nyman E, et al. *Spiroplasma ixodetis* infections in immunocompetent and immunosuppressed patients after tick exposure, Sweden. Emerg Infect Dis. 2022;28:1681–5. 35876734 10.3201/eid2808.212524 PMC9328919

[5] Matet A, Le Flèche-Matéos A, Doz F, Dureau P, Cassoux N. Ocular *Spiroplasma ixodetis* in newborns, France. Emerg Infect Dis. 2020;26:340–4. 31793858 10.3201/eid2602.191097 PMC6986854

[6] Aquilino A, Masiá M, López P, Galiana AJ, Tovar J, Andrés M, et al. First human systemic infection caused by *Spiroplasma.* J Clin Microbiol. 2015;53:719–21. 25428150 10.1128/JCM.02841-14 PMC4298541

[7] Lernout T, De Regge N, Tersago K, Fonville M, Suin V, Sprong H. Prevalence of pathogens in ticks collected from humans through citizen science in Belgium. Parasit Vectors. 2019;12:550. 31752967 10.1186/s13071-019-3806-z PMC6873681

[8] Madison-Antenucci S, Kramer LD, Gebhardt LL, Kauffman E. Emerging tick-borne diseases. Clin Microbiol Rev. 2020;33:e00083–18. 31896541 10.1128/CMR.00083-18 PMC6941843

[9] Wilhelmsson P, Lindblom P, Fryland L, Ernerudh J, Forsberg P, Lindgren PE. Prevalence, diversity, and load of *Borrelia* species in ticks that have fed on humans in regions of Sweden and Åland Islands, Finland with different Lyme borreliosis incidences. PLoS One. 2013;8:e81433. 24278437 10.1371/journal.pone.0081433 PMC3836827

[10] Krawczyk AI, Van Duijvendijk GL, Swart A, Heylen D, Jaarsma RI, Jacobs FH, et al. Effect of rodent density on tick and tick-borne pathogen populations: consequences for infectious disease risk. Parasit Vectors. 2020;13:1–17. 31959217 10.1186/s13071-020-3902-0 PMC6971888

[11] Gyllemark P, Wilhelmsson P, Elm C, Hoornstra D, Hovius JW, Johansson M, et al. Are other tick-borne infections overlooked in patients investigated for Lyme neuroborreliosis? A large retrospective study from South-eastern Sweden. Ticks Tick Borne Dis. 2021;12:101759. 34161869 10.1016/j.ttbdis.2021.101759

[12] Jaenson TG, Jaenson DG, Eisen L, Petersson E, Lindgren E. Changes in the geographical distribution and abundance of the tick *Ixodes ricinus* during the past 30 years in Sweden. Parasit Vectors. 2012;5:8. 22233771 10.1186/1756-3305-5-8 PMC3311093

[13] Carlströmer Berthén N, Tompa E, Olausson S, Nyberg C, Nyman D, Ringbom M, et al. The AxBioTick study: *Borrelia* species and tick-borne encephalitis virus in ticks, and clinical responses in tick-bitten individuals on the Aland Islands, Finland. Microorganisms. 2023;11:1100. 37317075 10.3390/microorganisms11051100 PMC10223457

[14] Strnad M, Hönig V, Růžek D, Grubhoffer L, Rego ROM. Europe-wide meta-analysis of Bo*rrelia burgdorferi* sensu lato prevalence in questing *Ixodes ricinus* ticks. Appl Environ Microbiol. 2017;83:e00609–17. 28550059 10.1128/AEM.00609-17 PMC5514677

