# Supplementary material for: Spiroplasma ixodetis in Ticks Removed from Humans, Sweden and Åland Islands, Finland
Source: Emerg Infect Dis. 2025 Nov;31(11):2159–62. doi: 10.3201/eid3111.250545 (PMC12704539; doi:10.3201/eid3111.250545)
Supplement: Appendix — Additional information about Spiroplasma ixodetis in ticks removed from humans, Sweden and Åland Islands, Finland. [file 25-0545-Techapp-s1.pdf]

Article DOI: https://doi.org/10.3201/eid3111.250545

EID cannot ensure accessibility for supplementary materials supplied by authors. Readers who have difficulty accessing supplementary content should contact the authors for assistance.

Spiroplasma ixodetis in Ticks Removed from Humans, Sweden and Åland Islands, Finland

Appendix

Tick Homogenization, Total Nucleic Acid Extraction, and Reverse Transcription of Nucleic Acid

Ticks were individually lysed and extracted for total nucleic acid (NA) using MagAttract RNA Tissue Mini M48 Kit (Qiagen, Hilden, Germany) and the BioRobot M48 Workstation (Qiagen) as previously described by Wilhelmsson et al. 2013 (*1*). NA from ticks collected from 2008–2009 underwent complementary DNA (cDNA) synthesizing using Illustra Ready-to-Go RT-PCR Bead kit (GE Healthcare, Amerham Place, UK), while ticks from 2010 were synthesized using the RevertAid First Strand cDNA synthesis kit (Thermo Fischer Scientific, Waltham, MA, USA) according to manufacturers instructions.: 15 µL extracted total NA, 2 µL Random Hexamer Primer, 8 µL 5X reaction Buffer, 2 µL RiboLock RNase Inhibitor (20 U/µL), 4 µL 10 mM dNTP mix, 2 µL RevertAid M-MuL V RT, and 7 µL RNase-free water to a final volume of 40 µL. The cDNA synthesizing was performed in the CGI-96 Palm-Cycler Thermal Cycler (Corbett Life Science, Sydney, Australia) according to the protocol: 5 min at 25°C, followed by 60 min at 42°C and 5 min at 70°C.

DNA Extraction of Human EDTA Plasma Samples

EDTA plasma samples (from both inclusion and follow-up visits) from participants with a removed tick containing *S. ixodetis* were analyzed for the presence of *S. ixodetis*. DNA was extracted using EZ1 & 2 DNA blood 200 µL kit (Qiagen) in an EZ1 Advanced XL Workstation (Qiagen) according to the manufacturer's instructions. Briefly, 200 µL EDTA plasma was used,

and each sample was eluted in 50 µL. The samples were analyzed immediately following the DNA extraction by real-time PCR.

Detection of *Spiroplasma ixodetis* by Real-Time PCR and Verification by Nucleotide Sequencing

Detection of *S. ixodetis* in ticks and EDTA plasma was carried out using a species-specific TaqMan real-time PCR assay (*2*). The primers Spir_rpoB-F (5′-TGTTGG ACCAAACGAAGTTG-3′), and Spir_rpoB-R (5′-CCAACAATTGGTGTTTGGGG-3′), and the probe Spir_rpoB-P (FAM-GCTAACCGTGCTTTAATGGG–MGB) are designed to target a 170-bp fragment of the RNA polymerase β subunit. Each sample was analyzed in a 20-µl reaction consisting of 10 µL Maxima® Probe qPCR Master Mix (2×) (Thermo Fisher Scientific), 0.4 µL of each primer (10 µM), 0.4 µL of the probe (10 µM) (Thermo Fisher Scientific), 6.8 µL RNase-free water (GE Healthcare), and 2 µL cDNA template. The PCR reactions were performed on a C1000 Thermal Cycler, CFX96 Real-Time PCR Detection System (Bio-Rad Laboratories, Inc., Hercules, CA, USA) using an activation step at 95°C for 5 min followed by 60 cycles of denaturation at 95°C for 5 s and annealing/extension at 60°C for 35 s, and ending with a cooling step for 37°C at 20 s. A positive control consisting of plasmid DNA spanning the nucleotides 108–278 of the DRET8 RNA polymerase β subunit gene (acc. no MG859277.1), synthesized and cloned in a pUC57 vector (Genscript USA Inc, Piscataway, NJ, USA), and a negative control consisting of RNase-free water (GE Healthcare) were included for each run. Confirmation analysis of the *S. ixodetis*-positive samples was performed by nucleotide sequencing of the amplified PCR products at Eurofins Genomics (Ebersberg, Germany), and all sequences obtained were confirmed by sequencing both strands. The electropherograms were first edited and analyzed using BioEdit Software v7.0 (Tom Hall, Ibis Therapeutics, Carlsbad, CA, USA), followed by a standard Basic Local Alignment Tool (BLAST) search against the GenBank database (https://blast.ncbi.nlm.nih.gov).

Statistical Analyses

The potential difference in the prevalence of *S. ixodetis* in different developmental stages, the geographic distribution of *S. ixodetis*-positive ticks in the four geographic regions, and the

co-existence between *S. ixodetis* and other tick-borne pathogens were evaluated using the Chi-square test. P-values ≤0.05 were considered statistically significant. Statistical analysis was performed using GraphPad Prism version 6.0 for Windows (GraphPad Software, Boston, MA, USA).

References

1. Wilhelmsson P, Lindblom P, Fryland L, Ernerudh J, Forsberg P, Lindgren PE. Prevalence, diversity, and load of *Borrelia* species in ticks that have fed on humans in regions of Sweden and Åland Islands, Finland with different Lyme borreliosis incidences. PLoS One. 2013;8:e81433. PubMed

2. Krawczyk AI, Van Duijvendijk GL, Swart A, Heylen D, Jaarsma RI, Jacobs FH, et al. Effect of rodent density on tick and tick-borne pathogen populations: consequences for infectious disease risk. Parasit Vectors. 2020;13:1–17 PubMed

Appendix Table 1. Primer and probe sequence and target gene for real-time PCR of *Anaplasma phagocytophilum*, *Babesia* spp., *Borrelia burgdorferi* sensu lato, *Borrelia miyamotoi*, *Neoehrlichia mikurensis*, *Rickettsia* spp, and tick-borne encephalitis virus

Agents	Function	Sequence (5′→ 3′)	Target gene
Anaplasma phagocytophilum	Forward	TTTTGGGCGCTGAATACGAT	*gltA*
	Reverse	TCTCGAGGGAATGATCTAATAACGT	
	Probe	VIC-TGCCTGAACAAGTTATG-MGBNFQ	
Babesia spp.	Forward	GTCTTGTAATTGGAATGATGG	*18S* rRNA
	Reverse	TAGTTTATGGTTAGGACTACG	
Borrelia burgdorferi sensu lato	Forward	GCT GAG TCA CGA AAG CGT AG	*16S* rRNA
	Reverse	CACTTAACACGTTAGCTTCGGTA	
	Probe	6-FAM-CGCTGTAAACGATGCACACTTGGT-MGB	
Borrelia miyamotoi	Forward	AGAAGGTGCTCAAGCAG	*flaB*
	Reverse	TCGATCTTTGAAAGTGACATA T	
	Probe	6-FAM-GCACAACAGGAGGGAGTTCAAGC-BHQ1–3	
Neoehrlichia mikurensis	Forward	CGGAAATAACAAAAGATGGA	*groEL*
	Reverse	ACCTCCTCGATTACTTTAG	
	Probe	6FAM-TTGGTGATGGAACTACA-MGB	
Rickettsia spp.	Forward	TCGCAAATGTTCACGGTACTTT	*gltA*
	Reverse	TCGTGCATTTCTTTCCATTGTG	
		6-FAM-TGCAATAGCAAGAACCGTAGGCTGGATG-MGBNFQ	
Tick-borne encephalitis virus (wild-type)		GGGCGGTTCTTGTTCTCC	11,054–11,121
	Reverse	ACACATCACCTCCTTGTCAGACT	
		6-FAM-TGAGCCACCATCACCCAGACACA-BHQ1	
Tick-borne encephalitis virus		GGCTTGTGAGGCAAAAAAGAA	1,329–1,416
		TCCCGTGTGTGGTTCGACTT	
	Probe	HEX-AAGCCACAGGACATGTGTACGACGCC-BHQ1	

Appendix Table 2. Results for *Spiroplasma ixodetis*–positive ticks (with sequencing results) including developmental stage, geographic distribution, blood feeding time, and real-time PCR results for coexistence of other tickborne pathogens*

Sample no.	Developmental stage	Region	Blood feeding time (h)	*Spiroplasma ixodetis* (species)	TBEV	*Borrelia* (species)	*Anaplasma phagocytophilum*	*Neoehrlichia mikurensis*	*Rickettsia* spp.	*Babesia* (species)
1	N	S.C.	53	Pos (*S. ixodetis*)	Neg	Neg	Neg	Neg	Neg	Neg
2	N	S.C.	43	Pos (*Spiroplasma* spp.)	Neg	Pos (*B. afzelii)*	Neg	Neg	Neg	Neg
3	N	S.C.	57	Pos (*Spiroplasma* spp.)	Neg	Neg	Neg	Neg	Neg	Neg
4	N	S.C.	26	Pos (*S. ixodetis*)	Neg	Neg	Neg	Neg	Neg	Neg
5	n.d.	S.C.	49	Pos (*S. ixodetis*)	Neg	Neg	Neg	Neg	Neg	Neg
6	A (m)	S.C.	44	Pos (*S. ixodetis*)	Neg	Neg	Neg	Neg	Neg	Neg
7	n.d.	S.C.	26	Pos (*S. ixodetis*)	Neg	Neg	Neg	Neg	Neg	Neg
8	N	S.M.	54	Pos (*S. ixodetis*)	Neg	Pos (*B. afzelii*)	Neg	Neg	Neg	Neg
9	N	S.M.	27	Pos (*Spiroplasma* spp.)	Neg	Neg	Neg	Neg	Neg	Neg
10	N	S.M.	57	Pos (*S. ixodetis*)	Neg	Neg	Neg	Neg	Neg	Neg
11	N	S.M.	<24	Pos (*S. ixodetis*)	Neg	Pos (*B. afzelii*)	Neg	Pos	Neg	Neg
12	N	S.M.	nd	Pos (*S. ixodetis*)	Neg	Pos (*B. afzelii*)	Neg	Neg	Neg	Neg
13	N	S.M.	<24	Pos (*S. ixodetis*)	Neg	Neg	Pos	Neg	Neg	Neg
14	N	S.M.	37	Pos (*S. ixodetis*)	Neg	Neg	Pos	Neg	Neg	Neg
15	N	S.M.	nd	Pos (*S. ixodetis*)	Neg	Pos (*B. afzelii*)	Neg	Neg	Neg	Neg
16	A (f)	S.C.	33	Pos (*S. ixodetis*)	Neg	Neg	Neg	Neg	Neg	Neg
17	A (f)	S.C.	35	Pos (*S. ixodetis*)	Neg	Neg	Neg	Neg	Neg	Neg
18	A (f)	S.C.	53	Pos (*S. ixodetis*)	Neg	Neg	Neg	Neg	Neg	Pos (*B. microti*)
19	N	S.M.	<24	Pos (*S. ixodetis*)	Neg	Neg	Neg	Neg	Neg	Neg
20	N	S.M.	31	Pos (*S. ixodetis*)	Neg	Pos (*B. afzelii*)	Neg	Neg	Neg	Neg
21	N	S.M.	<24	Pos (*S. ixodetis*)	Neg	Pos (*B. garinii*)	Neg	Neg	o.s.	Neg
22	N	S.C.	nd	Pos (*S. ixodetis*)	Neg	Pos (*B. valaisiana*)	Neg	Neg	Neg	Neg
23	A (f)	S.C.	45	Pos (*S. ixodetis*)	Neg	Neg	Neg	Neg	Neg	Neg
24	N	S.C.	<24	Pos (*S. ixodetis*)	Neg	Neg	Neg	Neg	Neg	Neg
25	N	S.C.	40	Pos (*S. ixodetis*)	Neg	Neg	Neg	Neg	Neg	Neg
26	N	S.C.	nd	Pos (*S. ixodetis*)	Neg	Neg	Neg	Pos	Neg	Neg
27	N	S.M.	>60	Pos (*Spiroplasma* spp.)	Neg	Neg	Neg	Neg	Neg	Neg
28	A (f)	S.M.	34	Pos (*S. ixodetis*)	Neg	Neg	Neg	Neg	Pos	Neg
29	N	S.C.	<24	Pos (*S. ixodetis*)	Neg	Neg	Neg	Neg	Neg	Neg
30	N	S.C.	30	Pos (*S. ixodetis*)	Neg	Neg	Neg	Neg	Neg	Neg
31	N	S.C.	nd	Pos (*Spiroplasma* spp.)	Neg	Pos (*B. afzelii*)	Neg	Pos	Neg	Neg
32	N	S.C.	43	Pos (*S. ixodetis*)	Neg	Neg	Neg	Neg	Neg	Neg
33	N	S.C.	33	Pos (*S. ixodetis*)	Neg	Neg	Neg	Neg	Neg	Neg
34	N	S.C.	39	Pos (*S. ixodetis*)	Neg	Neg	Neg	Neg	Neg	Neg
35	N	S.C.	38	Pos (*Spiroplasma* spp.)	Neg	Neg	Neg	Neg	Neg	Neg
36	A (f)	S.C.	21	Pos (*Spiroplasma* spp.)	Neg	Neg	Neg	Neg	Neg	Neg
37	N	S.C.	nd	Pos (*S. ixodetis*)	Neg	Neg	Neg	Neg	o.s.	Neg
38	A (f)	S.M.	33	Pos (*S. ixodetis*)	Neg	Neg	Neg	Neg	Neg	Neg
39	A (f)	S.M.	37	Pos (*S. ixodetis*)	Neg	Pos (*B. valaisiana*)	Neg	Neg	Neg	Neg
40	N	S.M.	<24	Pos (*S. ixodetis*)	Neg	Neg	Neg	Neg	Neg	Neg
41	A (f)	S.M.	<24	Pos (*S. ixodetis*)	Neg	Neg	Neg	o.s.	o.s.	o.s.
42	N	S.M.	33	Pos (*S. ixodetis*)	Neg	Neg	Neg	Neg	Neg	Neg
43	N	S.C.	<24	Pos (*S. ixodetis*)	Neg	Neg	Neg	Pos	Neg	Neg
44	N	S.C.	<24	Pos (*S. ixodetis*)	Neg	Neg	Neg	Neg	Neg	Neg

Sample no.	Developmental stage	Region	Blood feeding time (h)	*Spiroplasma ixodetis* (species)	TBEV	*Borrelia* (species)	*Anaplasma phagocytophilum*	*Neoehrlichia mikurensis*	*Rickettsia* spp.	*Babesia* (species)
45	N	S.C.	52	Pos (*S. ixodetis*)	Neg	Neg	Neg	Neg	Neg	Neg
46	N	S.C.	nd	Pos (*Spiroplasma* spp.)	Neg	Neg	Neg	Neg	Pos	Neg
47	A (f)	S.C.	<24	Pos (*S. ixodetis*)	Neg	Pos (*B. valaisiana*)	Neg	Neg	Neg	Neg
48	A (f)	S.C.	nd	Pos (*Spiroplasma* spp.)	Neg	Neg	Neg	Neg	Neg	Neg
49	A (f)	S.C.	nd	Pos (*S. ixodetis*)	Neg	Pos (*B. afzelii*)	Neg	Neg	Neg	Neg
50	N	S.M.	<24	Pos (*S. ixodetis*)	Neg	Neg	Neg	Neg	o.s.	Neg
51	A (f)	S.M.	<24	Pos (*S. ixodetis*)	Neg	Neg	Neg	Neg	Neg	Neg
52	A (f)	S.M.	<24	Pos (*S. ixodetis*)	Neg	Pos (*B. valaisiana*)	Neg	Neg	Neg	Neg
53	A (f)	S.M.	>72	Pos (*S. ixodetis*)	Neg	Pos (*Borrelia* spp.)	Neg	Pos	Neg	Neg
54	L	S.M.	nd	Pos (*Spiroplasma* spp.)	Neg	Neg	Neg	Neg	Neg	Neg
55	N	S.M.	82	Pos (*S. ixodetis*)	Neg	Neg	Neg	Neg	Neg	Neg
56	A (f)	S.M.	53	Pos (*S. ixodetis*)	Neg	Neg	Neg	Neg	Pos	Neg
57	N	S.M.	43	Pos (*S. ixodetis*)	Neg	Neg	Neg	Neg	Neg	Neg
58	N	S.M.	57	Pos (*Spiroplasma* spp.)	Neg	Neg	Neg	Neg	o.s.	Neg
59	N	S.M.	26	Pos (*S. ixodetis*)	Neg	Neg	Neg	Neg	Neg	Neg
60	A (f)	S.M.	49	Pos (*S. ixodetis*)	Neg	Neg	Neg	Neg	Neg	Neg
61	N	S.M.	44	Pos (*S. ixodetis*)	Neg	Neg	Pos	Neg	Neg	Neg
62	N	S.M.	26	Pos (*S. ixodetis*)	Neg	Neg	Neg	Neg	Neg	Neg
63	L	Å	54	Pos (*S. ixodetis*)	Neg	Neg	Neg	Neg	Neg	Neg
64	A (f)	Å	27	Pos (*S. ixodetis*)	Neg	Neg	Neg	Neg	Neg	Neg
65	L	Å	57	Pos (*Spiroplasma* spp.)	Neg	Neg	Neg	Neg	Neg	Neg
66	A (f)	Å	<24	Pos (*S. ixodetis*)	Neg	Pos (*Borrelia* spp.)	Neg	Neg	Neg	Neg
67	N	S.C.	nd	Pos (*S. ixodetis*)	Neg	Neg	Neg	Neg	Neg	Neg
68	N	S.C.	<24	Pos (*S. ixodetis*)	Neg	Neg	Neg	Neg	o.s.	Neg
69	N	S.C.	37	Pos (*S. ixodetis*)	Neg	Neg	Neg	Neg	Neg	Neg
70	A (f)	S.C.	nd	Pos (*S. ixodetis*)	Neg	Pos (*Borrelia* spp.)	Neg	Neg	Neg	Neg
71	n.d.	S.C.	33	Pos (*S. ixodetis*)	Neg	Neg	Neg	Neg	Neg	Neg
72	A (f)	S.C.	35	Pos (*S. ixodetis*)	Neg	Pos (*B. afzelii*)	Neg	Neg	Neg	Neg

*All PCR reactions were performed on a C1000 Thermal Cycler, CFX96 Real-Time PCR Detection System (Bio-Rad Laboratories, Inc.). Samples marked with spp. could not be sequenced, probably due to high cycle threshold values. Å, Åland Islands, Finland; A (f), adult female, A (m), adult male, L, larvae; N, nymph, n.d., not determined, Neg, negative; o.s., out of sample, Pos, positive; S.C., southcentral; S.M., southernmost; TBEV, tick-borne encephalitis virus.
